# The human epididymis protein 4 acts as a prognostic factor and promotes progression of gastric cancer

**DOI:** 10.1007/s13277-014-2858-0

**Published:** 2014-11-29

**Authors:** Yun-Di Guo, Jing-Hao Wang, Huan Lu, Xiao-Ning Li, Wei-Wei Song, Xiao-Dan Zhang, Wen-Ming Zhang

**Affiliations:** 1 Suzhou Health College, Suzhou, 215009 China; 2 Department of Endoscopy, Fudan University Shanghai Cancer Center, Shanghai, 200032 China; 3 Department of Oncology, Shanghai Medical College, Fudan University, Shanghai, 200032 China; 4 Department of Obstetrics and Gynecology, Fengxian Hospital, Southern Medical University, 201499 Shanghai, China; 5 Department of Obstetrics and Gynecology, Shanghai Fengxian District Central Hospital, Shanghai, 201400 China

**Keywords:** HE4, Gastric cancer, Apoptosis, Proliferation, Migration

## Abstract

Human epididymis protein 4 (HE4), represented as an epididymis-specific gene and designated a WAP four-disulfide core domain protein 2 (WFDC2), is amplified in many tumors. However, little is known about its clinical significance and biological function in gastric carcinomas. We found that HE4 was more commonly observed in gastric carcinoma tissues than in normal tissues and was significantly correlated with Lauren classification, TNM stage, and tumor size by immunohistochemistry. The overall survival rate of patients with HE4 low expression was significantly higher than that of the patients with HE4 high expression. In addition, silencing of HE4 expression inhibits cell proliferation and migration and enhances cell apoptosis. Subsequent studies reveal that the Src, Akt, and Erk1/2 signaling may be involved with pro-survival and anti-apoptotic effects of the HE4 on gastric cancer cells. Taken together, this study provides new evidence on promotive effects of the HE4 on gastric cancer progression and indicates that HE4 might be a promising prognostic factor for gastric cancer diagnosis.

## Introduction

Globally, gastric cancer is one of the most common malignancies [1] and the leading cause of cancer-related death in China [2]. Despite the trend for decreasing incidence, the 5-year survival rate of gastric carcinoma is approximately 27 % for patients from 2001 to 2007 [3]. Additionally, some chemotherapeutic drugs have no ideal curative effects and furthermore have many unexpected side effects. Therefore, it is essential to further explore the molecular mechanisms of development and progression of gastric carcinoma and expect specific and sensitive markers with valuable and early diagnosis.

Human epididymis protein 4 (HE4) was found as a marker of cancer recently and has been reported in multiple types of neoplasia [4, 5] such as ovarian cancer [5], endometrial cancer [1], lung cancer [6], and breast cancer [7], whereas there are no reports about HE4 on clinicopathological significance, cellular function, and mechanism in gastric carcinoma.

In our study, we ascertained that the HE4 was significantly upregulated in human gastric cancer and correlated with Lauren classification, TNM stage, and tumor size, and the overall survival rate of patients without HE4 expression was significantly higher than the rate of those with HE4 overexpression. In addition, we characterized that silencing the HE4 inhibited proliferation and migration and enhanced apoptosis. Furthermore, we found that the HE4 might regulate proliferation, migration, and apoptosis through Src/Fak, Akt, and Erk1/2 signaling in gastric cancer cells. In conclusion, our findings illuminate that HE4 might be a new therapeutic value marker for gastric cancer.

## Materials and methods

### Cell culture

The human gastric cancer cell lines (MKN-28, MKN-45, MGC-803, NCI-N87, and HGC-27) were obtained from the American Type Culture Collection. All cell lines were grown at 37 °C in 5 % CO_2_ in RPMI 1640 medium (Gibco) or Dulbecco’s modified Eagle’s medium (Gibco) with 10 % fetal bovine serum (Gibco).

### Patients’ samples

A total of 250 gastric carcinomas were consecutively collected from surgical resection at Shanghai Cancer Center between 1 January 2005 and 31 December 2008. None of the patients with carcinomas (31–86 years, mean = 62 years) underwent either chemotherapy or radiotherapy before surgery. All tumor tissue specimens were obtained with informed consent for clinical research, and protocols were approved by the ethical review committee of the World Health Organization Collaborating Center for Research in Human Production (authorized by the Shanghai Municipal Government). All patients were followed up by consulting their documents and through telephone interviews.

### Quantitative reverse transcriptase-PCR

According to the manufacturer’s instruction, total RNA was abstract by TRIzol reagent (Invitrogen). Complementary DNA was synthesized using Prime Script RT Reagent Kit (TaKaRa). The expression of HE4 was evaluated by quantitative reverse transcriptase-PCR (q-PCR) in an ABI7500 system using SYBR Green PCR Master Mix Reagents Kit (TaKaRa). Glyceraldehyde-3-phosphate dehydrogenase (GAPDH) was used as an internal control.

### siRNA interference

Three synthetic double-stranded siRNAs, which target the HE4, were transfected into MKN-28 cells and MGC-803 cells, respectively, with RNAi Mate Transfection Agent (GenePharma) for 48 h. The cells were considered as cells transient knockdown HE4 and verified by quantitative real-time PCR and Western blotting analysis.

### Western blot analysis

Cell protein lysates were denatured by boiling for 5 min and separated by 4–12 % SDS-PAGE and transferred to nitrocellulose membranes. After blocking with 5 % skimmed milk for 1 h at room temperature, membranes were then incubated with primary antibodies: anti-HE4 (Abcam), anti-p-AKT, anti-AKT, anti-p-Erk, anti-Erk, anti-p-FAK, anti-FAK, anti-p-Src and anti-Src (Cell signaling), and GAPDH (Sigma) overnight at 4 °C. Horseradish peroxidase-labeled goat anti-rabbit (1:1000) or goat anti-mouse (1:5000) IgG (Proteintech) was incubated for 1 h at room temperature. Specific protein bands were developed using the Odyssey imaging system (LI-COR).

### Tissue microarrays

Core tissue biopsy specimens (diameter 2 mm) were obtained from individual paraffin-embedded gastric carcinomas (donor blocks) and arranged in new recipient paraffin blocks (tissue assay blocks). Nonneoplastic gastric mucosa specimens were included in each of the assay blocks.

### Immunohistochemistry

Gastric carcinoma tissue arrays were first incubated using the antibody for HE4. The sections were immunostained with primary anti-HE4 (1:50 Abcam) and a universal biotinylated secondary antibody was developed. Scoring was conducted according to the ratio and intensity of positive-staining cells: 0–10 % scored 0, 11–30 % scored 1, 31–60 % scored 2, and 61–100 % scored 3. Then scored 0–1 was designated as low expression and scored 2–3 as high expression.

### Migration assay

Cells were seeded in triplicate in 24-well Transwell plates (Coster) for 24 h. Then, the migratory cells on the lower surface were fixed with methanol and stained with hematoxylin. For quantification, cells were counted under a microscope in five predetermined fields.

### Proliferation assay

Cells were seeded in 96-well plates at 3 × 10^3^ cells per well. Five independent experiments were performed in triplicate. According to the manufacturer’s protocol, Cell Counting Kit-8 (CCK8, Dojindo, Japan) was used to measure cell viability for 4 days. And cell viability was obtained by measuring the absorbance at 450 nm wavelength using Power Wave XS microplate reader (BIO-TEK).

### Flow cytometry

Apoptotic cells were analyzed using Annexin V-FITC and PI (BD Biosciences Clontech). Cells were trypsinized, washed twice with 4 °C PBS, and then resuspended in 1× binding buffer at 1 × 106 cells/ml. Each of the cells were washed with 1× PBS and stained with 200 μl binding buffer containing 3.5 μl Annexin V and 3.5 μl propidium iodide (PI), incubated at room temperature for 20 min, and analyzed using flow cytometry (BD Biosciences Clontech).

### Statistical analysis

The SPSS 16.0 program (SPSS, Chicago, IL, USA) was used for statistic analysis. Kaplan-Meier survival plots were generated, and comparisons between the survival curves were made with the log-rank test. The chi-square test and Student’s *t* test were used for comparison between groups. All data were shown as mean ± SD. *P* < 0.05 was considered statistically significant.

## Results

### HE4 is frequently upregulated in gastric cancer tissues

**Fig. 1:**
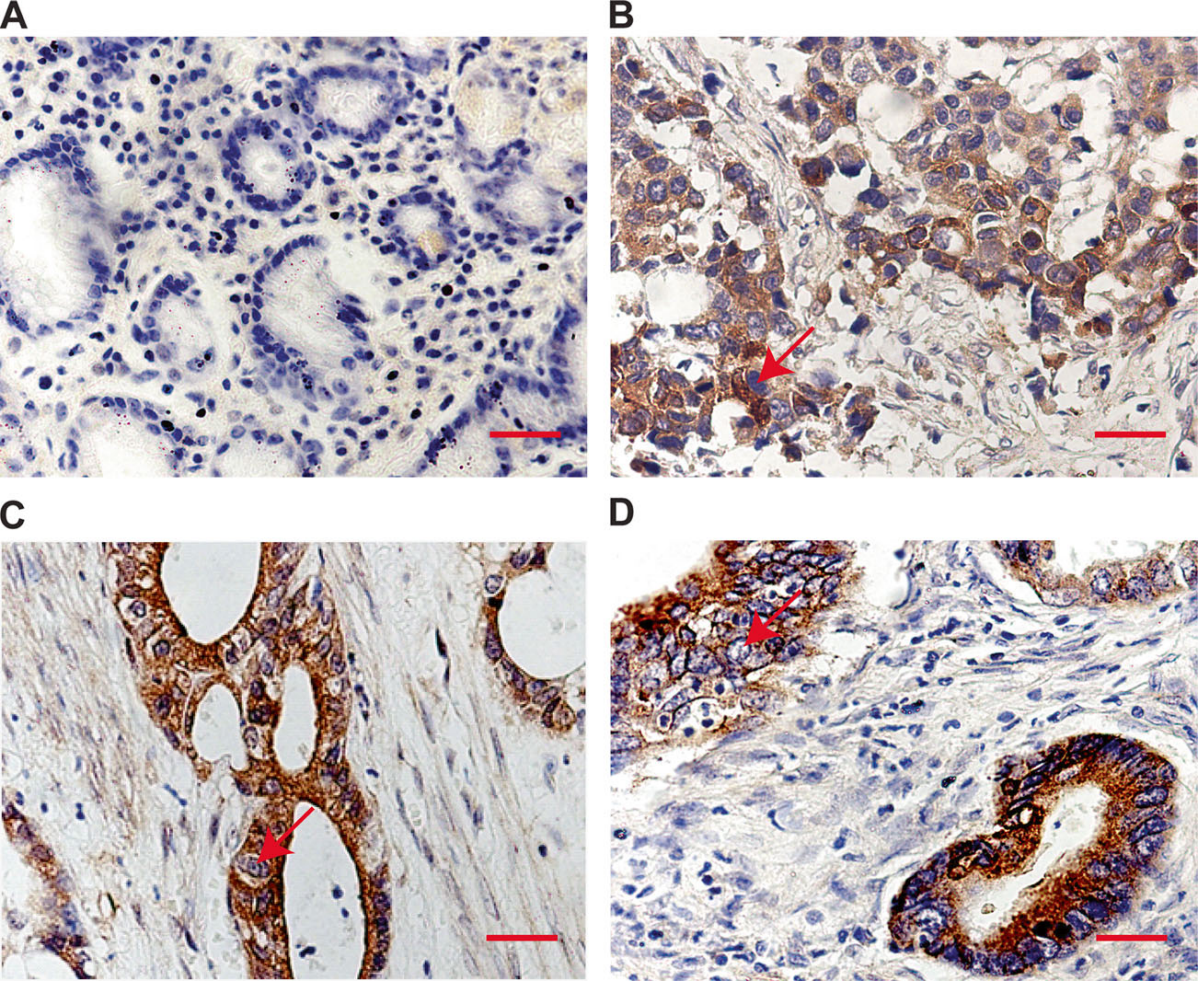
HE4 is frequently upregulated in gastric cancer tissues. Representative photomicrographs of the HE4 immunoreactivity in normal gastric mucosa (**a**), poorly differentiated adenocarcinoma (**b**), moderately differentiated adenocarcinoma (**c**), and well-differentiated adenocarcinoma (**d**). The *arrows* represent HE4 positive staining without reaction in the nuclei (*scale bar*: 50 μm)

To study the discrepancies of HE4 in gastric carcinoma, we detected the expressions of HE4 on the gastric cancer tissue array using immunohistochemical analysis. In 243 cases of normal gastric mucosa, weak nuclear staining was identified in only 11 cases for HE4 with 4.53 % (11/243). However, strong expression of HE4 was identified in 62.96 % (153/243) of gastric carcinoma patients. There was statistical significance between the expressions of HE4 in normal gastric mucosa (Fig. 1a) and gastric carcinoma (Fig. 1b–d). Collectively, these findings suggest that HE4 is upregulated in gastric cancer tissues.

### HE4 expression is closely related to Lauren classification, TNM stage, tumor size, and patient prognosis in gastric cancer

In 243 cases of gastric carcinoma, the HE4 expression was not significantly related to patient age, gender, Japanese classification, tumor stage, lymph node metastasis and tumor invasion (*P* > 0.05). Nevertheless, the expression of HE4 was significantly correlated with Lauren classification (*P* = 0.043), TNM stage (*P* = 0.020), and tumor size (*P* = 0.042) (Table 1).

**Table 1:** Statistic analysis of HE4 with patients’ clinicopathologic parameters

Characteristics		HE4 expression level				*χ* ^2^	*P* value
		Negative	Low	Middle	High		
Age (years)	<65	51	47	22	16	0.921	0.820
	≥65	39	41	18	9		
Sex	Female	67	58	28	16	1.936	0.586
	Male	23	30	12	9		
Lauren’s classification	Intestinal	39	21	15	7	8.163	0.043
	Diffuse	51	67	25	18		
Histologic grade	Pap	1	2	1	1	12.774	0.805
	Tub1	3	2	1	0		
	Tub2	32	30	18	8		
	Por1	46	42	16	14		
	Por2	0	1	0	0		
	Muc	2	2	3	2		
	Sig	4	7	1	0		
Depth of invasion	T1	20	12	4	2	10.356	0.322
	T2	12	15	8	2		
	T3	51	48	21	18		
	T4	7	13	7	3		
TNM stage	I	26	19	7	3	19.671	0.020
	II	23	29	12	4		
	III	33	36	19	11		
	IV	8	4	2	7		
Lymph node metastasis	Positive	47	44	23	17	2.850	0.415
	Negative	43	44	17	8		
Lymph	Yes	13	5	8	2	6.822	0.078
	No	77	83	32	23		
Tumor size	<4 cm	46	34	13	6	8.198	0.042
	≥4 cm	44	54	27	19		
Differentiation	Well	9	10	3	2	7.828	0.251
	Moderate	27	26	21	8		
	Poor	54	52	16	15		

*Pap* papillary adenocarcinoma, *Tub1* well-differentiated tubular adenocarcinoma, *Tub2* moderately differentiated tubular adenocarcinoma, *Por1* solid poorly differentiated adenocarcinoma, *Por2* non-solid poorly differentiated adenocarcinoma, *Muc* mucinous adenocarcinoma, *Sig* signet-ring cell carcinoma

**Fig. 2:**
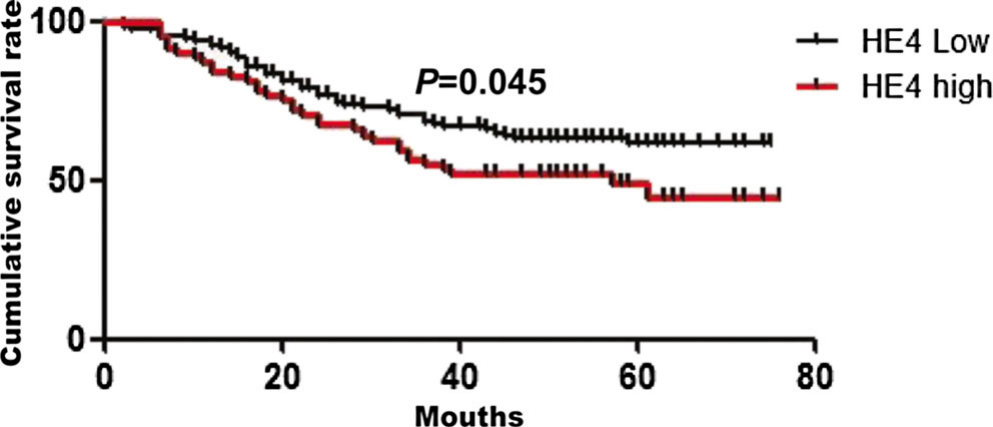
The relationship between expression of HE4 protein and patients’ prognosis. The overall survival rate of patients with HE4 low expression, as determined by the log-rank test, was significantly higher than the rate of those with HE4 high expression (*P* = 0.045)

Next, we observed that the mean duration of follow-up was 47 months after surgery and 75th percentile of duration was 55 months. During the follow-up period, 97 of the 243 patients (39.9 %) died. The overall survival rate of patients with HE4 overexpression, as determined by the log-rank test, was significantly lower than the rate of those without HE4 expression (*P* = 0.045) (Fig. 2).

### Silencing of HE4 expression enhances apoptosis and inhibits proliferation

**Fig. 3:**
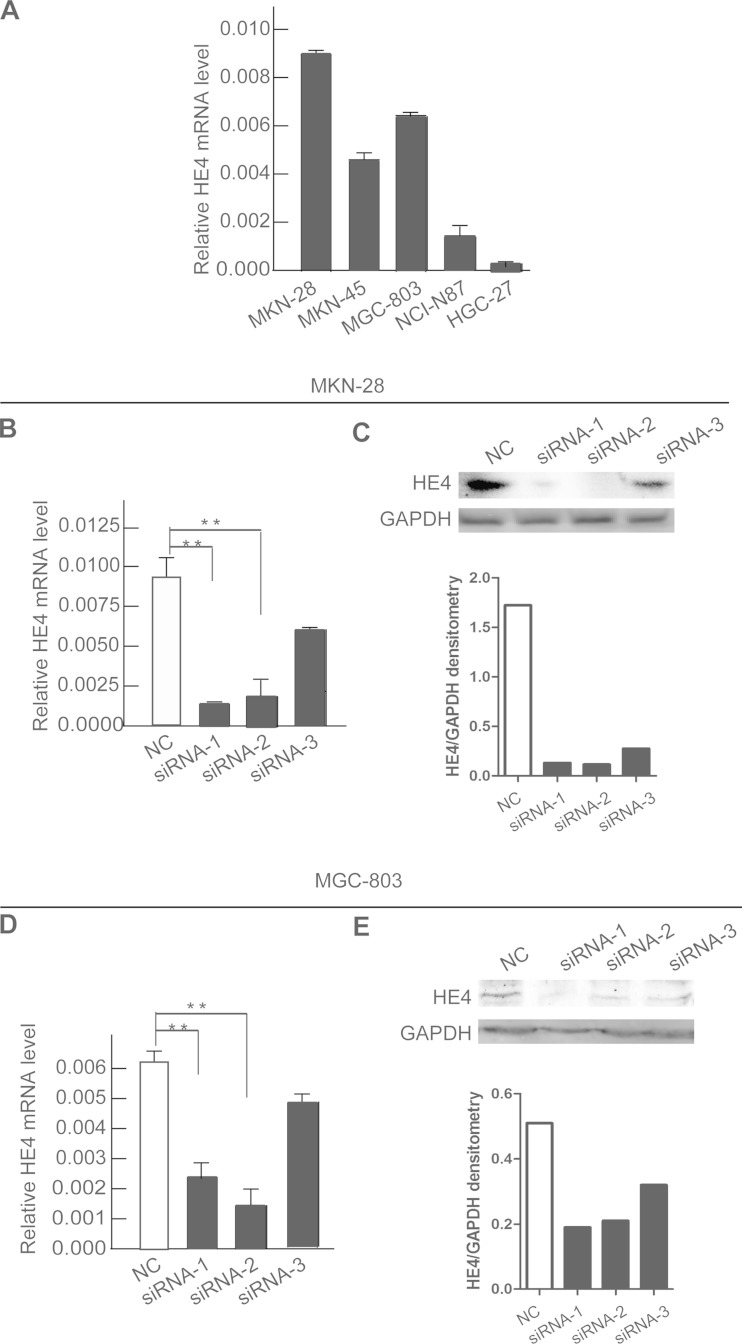
The expression level of HE4 in different gastric cancer cells lines and validation of siRNA interference efficiency. **a** MKN-28 and MGC-803 are higher than other gastric cancer cells in the mRNA level of HE4. The siRNA1 and siRNA2 could notably restrain HE4 by real-time PCR (**b**) and Western blotting (**c**) in MKN-28 cells. The siRNA1 and siRNA2 could notably restrain HE4 by real-time PCR (**d**) and Western blotting (**e**) in MGC-803 cells. (sh-NC versus sh-1 or sh-2, *P < 0.05; **P < 0.01)

Emerging evidences have shown that HE4 is upregulated in ovarian, endometrial, lung, and breast cancer, etc. [1, 5–7]; we thus examined the mRNA level of HE4 in gastric cancer cells by quantitative real-time PCR. Our data identified that HE4 was upregulated in a panel of gastric cancer cell lines (MKN-28, MKN-45, MGC-803, NCI-N87, and HGC-27) (Fig. 3a).

To determined whether HE4 protein are responsible for gastric cancer progression, we used three different small interfering RNAs to interfere HE4 in gastric cancer cells MKN-28 and MGC-803, which are higher in the mRNA level of HE4. Then, we examined the mRNA level of the siRNAs in MKN-28 and MGC-803, respectively. We found that the mRNA level of siRNA1, siRNA2, and siRNA3 was 14.3, 22.2, and 66.8 % in gastric cancer cell MKN-28 (Fig. 3b), while the mRNA level of siRNA1, siRNA2, and siRNA3 was 37.6, 24.7, and 44.2 % in gastric cancer cell MGC-803 (Fig. 3c). Similarly, the expression pattern of siRNAs in gastric cancer cells MKN-28 and MGC-803 consisted with the mRNA level (Fig. 3b, c). All the data indicated that siRNA1 and siRNA2 could notably restrain HE4, which were considered statistically significant. Therefore, siRNA1 and siRNA2 were chosen for the follow-up tests.

**Fig. 4:**
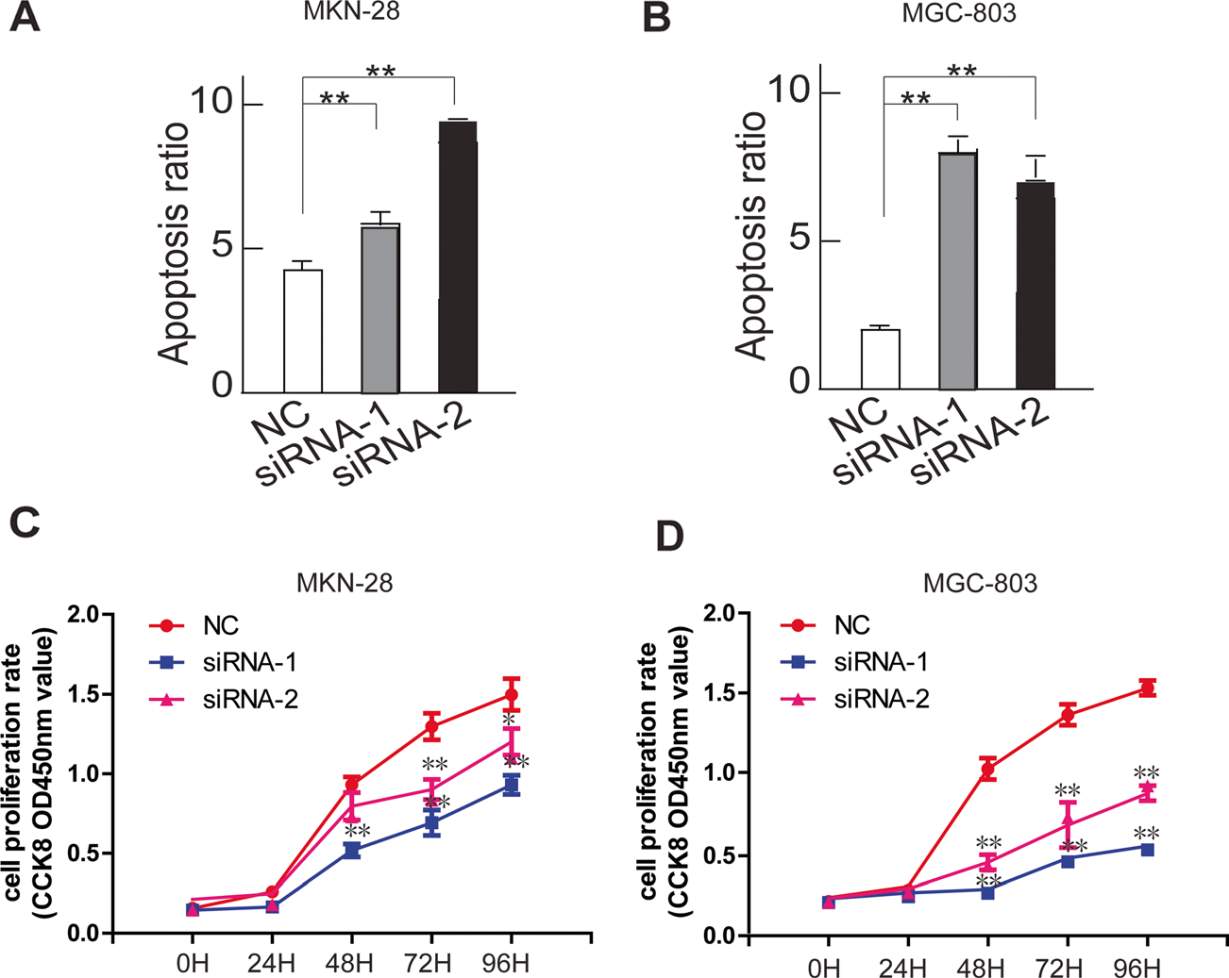
Silencing of HE4 enhances apoptosis and inhibits proliferation in gastric cancer cells. Apoptosis ratio of MKN-28 (**a**) and MGC-803 (**b**) cells were analyzed by Annexin V and PI staining and flow cytometric analysis (sh-NC versus sh-1 or sh-2, *P < 0.05; **P < 0.01). Statistic data shown right are means ± SD of apoptotic cell rates from triplicate samples. Data are representative of three independent experiments. Effect of HE4 knockdown on cell survival of MKN-28 (**c**) and MGC-803 (**d**) cells with serum deprivation were analyzed by CCK8 assay (sh-NC versus sh-1 and sh-NC versus sh-2, *P < 0.05; **P < 0.01)

In MKN-28 and MGC-803 cells treated with a HE4-specific siRNA1 and siRNA2, the number of early and late apoptotic cells was significantly higher than control group, after the cells were starved for 24 h. We conclude that the deficient HE4 expression level promotes apoptosis in gastric cancer cells (Fig. 4a, b).

Because HE4 expression was significantly correlated with tumor size mentioned above, we first measured the cell viability using CCK8 reagent and found that suppressive effect on the cell growth was not an immediate cell response in MKN-28 and MGC-803 cells treated with a HE4-specific siRNA1 and siRNA2; rather, it took more than 24 h to become prominent (Fig. 4c, d). We infer from these results that gastric cancer cells with low HE4 expression levels have a growth disadvantage.

### Silencing of HE4 expression inhibits migration

**Fig. 5:**
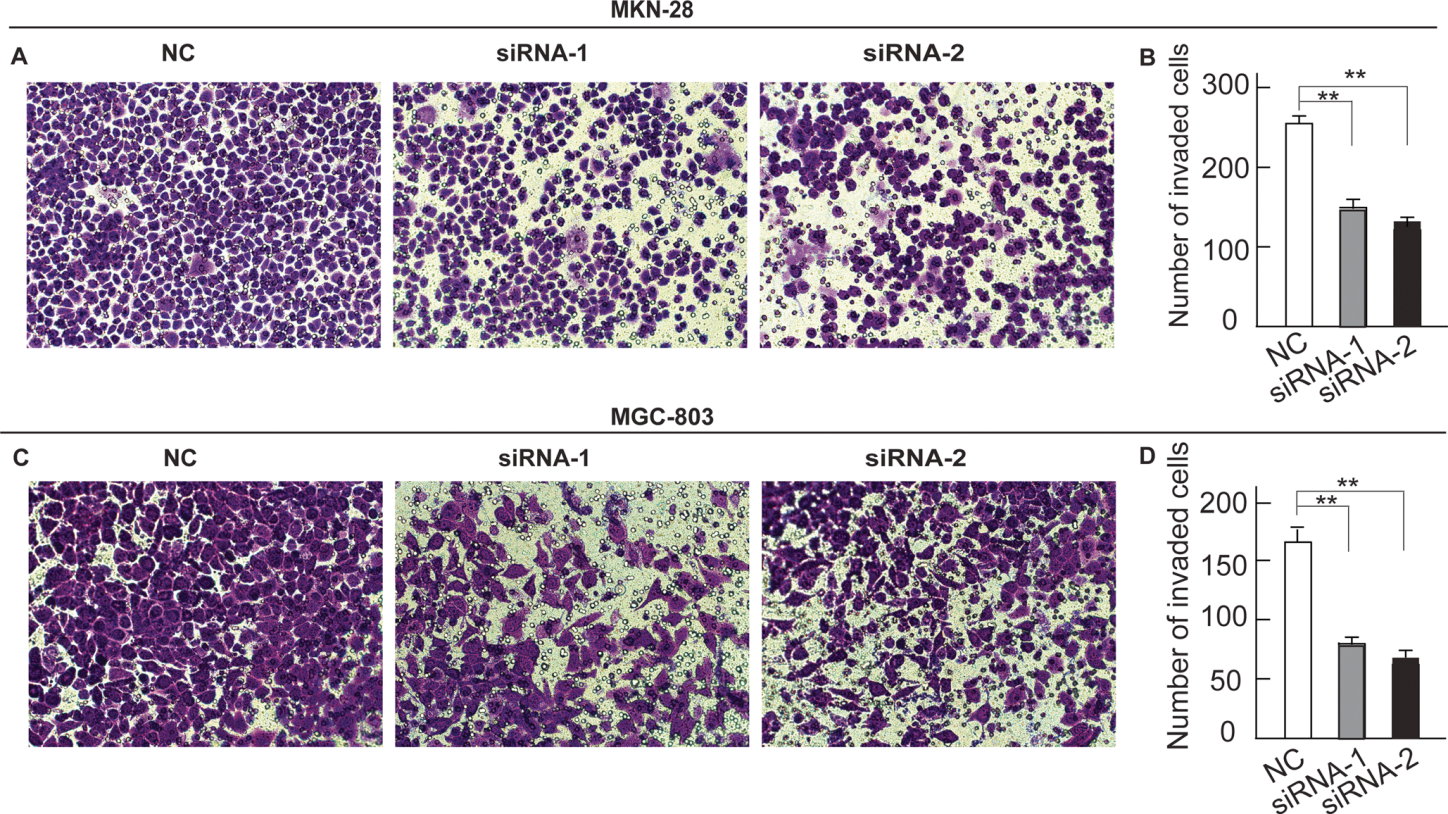
Silencing of HE4 inhibits gastric cancer cells migration. MKN-28 cells (**a**, **b**) and MGC-803 (**c**, **d**) cells that migrated through the transwell chambers and photographed at ×100 magnification. The in vitro migration ability of MKN-28 cells (**a**, **b**) and MGC-803 (**c**, **d**) was measured by determining the number of uncoated cells that penetrated through the transwell chambers (*columns* mean values, *bar* SD; *P < 0.05; **P < 0.01)

Next, to investigate the effects of downregulating HE4 on migration, we conducted in vitro migration assays to evaluate the migration ability of MKN-28 and MGC-803 cells. MKN-28 cells treated with a HE4-specific siRNA1 and siRNA2 decreased averagely in migration of 56.7 % (144.0 ± 15.0 cells versus 253.8 ± 15.6 cells per field, respectively; *P* < 0.05) and 50.0 % compared to the control cells (127.1 ± 5.9 cells versus 253.8 ± 15.6 cells per field, respectively; *P* < 0.05) (Fig. 5a). Furthermore, MGC-803 cells treated with a HE4-specific siRNA1 and siRNA2 decreased averagely in migration of 48.6 % (79.7 ± 7.4 cells versus 164.0 ± 14.6 cells per field, respectively; *P* < 0.05) and 38.5 % compared to the control cells (164.0 ± 14.6 cells versus 63.2 ± 10.6 cells per field, respectively; *P* < 0.05) (Fig. 5b). These results suggest that HE4 expression is associated with the migration potential of gastric cell lines in vitro.

### Silencing of HE4 affects Src, Akt, and Erk1/2 signaling in gastric cancer cells

**Fig. 6:**
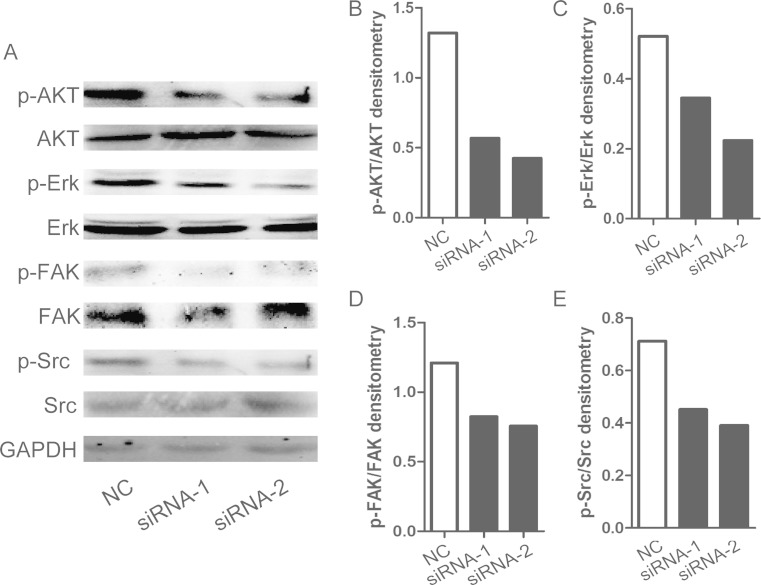
**a**–**e** Silencing of HE4 inhibits activation of ERK1/2 and AKT. Analysis of activation of Akt, Erk1/2, Fak, Src in HE4 knockdown MKN-28, and MGC-803 cells using GAPDH as a loading control

We were interested in how HE4 regulates proliferation, migration, and apoptosis; hence, we further explored the intracellular signaling. The results showed that silencing HE4 could significantly diminish the phosphorylation level of Akt, Erk1/2, Fak, and Src which play important roles in regulating cell proliferation, migration, and apoptosis [16]. The data facilitates that the identification of HE4 regulates proliferation, migration, and apoptosis through Src/Fak, Akt, and Erk1/2 signaling in gastric cancer cells (Fig. 6a–e).

## Discussion

Gastric cancer is the fourth most common carcinomas worldwide; however, its incidence and mortality varies geographically [8]. In China, the mortality rate of gastric cancer ranked the third in overall cancer mortality [9].

The HE4 (WFDC2) encodes a protein which has a WAP-type four-disulfide core domain [10] and was initially found in the distal epididymis epithelium [5, 11]. Hellstrom et al. reported that the quantitation of the HE4 protein levels in serum was more frequently positive than that of the CA125 levels in ovarian cancer; therefore, it could be used as a biomarker of ovarian cancer [5]. And their subsequent study confirmed the observation [12]. Likewise, study from Moore et al. showed that the HE4 was elevated at all stage of the endometrial cancer by examining the levels of the HE4 in the pre-operative serum samples from surgically staged patients with endometrioid adenocarcinoma of the uterus [13]. Recently, the HE4 has been found to be differently expressed in various cancers such as ovarian cancer [5], endometrial cancer [1], lung cancer [6], breast cancer [7], etc.

Kristjansdottir et al. used a cohort of 373 patient blood samples from women scheduled for surgery for a malignant suspicious cystic ovarian mass and showed the significant variations of HE4 and CA125 levels within different tumors and histotypes, and the highest levels of HE4 and CA125 were in the more aggressive and genetically highly instable tumors [14]. In accordance with our study, we discovered that the HE4 expression was significantly correlated with Lauren classification (*P* = 0.043), TNM stage (*P* = 0.020), and tumor size (*P* = 0.042) (Table 1). Furthermore, we found that the overall survival rate of the patients without the HE4 expression was significantly higher than the rate of those with the HE4 overexpression. These results were in line with the previous studies that the 5-year overall survival rate was lower in the HE4-positive group than that in the HE4-negative group in pulmonary adenocarcinoma [15].

To further explore the molecular mechanisms of the development and progression of gastric carcinoma, we explored the functions of the HE4 in the gastric cancer cell lines and tissues. As expected, we found that the repression of the HE4 expression inhibited proliferation and migration and enhanced apoptosis. Collectively, these observations indicated that the HE4 might play an important regulatory role in the development and progression of gastric carcinoma.

Moreover, recent studies have shown that the levels of phosphorylated Akt and ERK1/2 regulate various cell functions, such as angiogenesis, immigration, and survival [16]. Therefore, we explored the intracellular signaling and found that silencing the HE4 could significantly diminish the phosphorylation level of Akt, Erk1/2, Fak, and Src. These results facilitate the identification of the HE4 regulating proliferation, migration, and apoptosis through Src/Fak, Akt, and Erk1/2 signaling in gastric cancer cells.

In conclusion, the HE4 in our study of the gastric cancer is applicable for histopathologic diagnosis and might be a new target for therapeutic interventions. We will amplify samples of sera from patients with gastric cancer to verify our observation and explore the intracellular signaling in further study.
